# 超声提取-气相色谱-质谱法测定本草香中16种颗粒态多环芳烃含量及其排放特征分析

**DOI:** 10.3724/SP.J.1123.2022.01022

**Published:** 2022-12-08

**Authors:** Meizhen CAI

**Affiliations:** 国家燃香类产品质量监督检验中心(福建), 福建 泉州 362600; National Quality Supervision and Inspection Center for Incense Products (Fujian), Quanzhou 362600, China

**Keywords:** 超声提取, 气相色谱-串联质谱, 多环芳烃, 颗粒态, 排放特征, 特征比值, 本草香, ultrasonic extraction, gas chromatography-mass spectrometry (GC-MS), polycyclic aromatic hydrocarbons (PAHs), particle-phase, emission characteristics, characteristic ratio, herbal incense

## Abstract

本草香颗粒态多环芳烃(PAHs)的分析对探究人体健康和环境安全的影响具有重要意义,但目前相关研究主要针对竹签香,对于配方更为复杂、日常使用更为频繁的本草香颗粒态PAHs定量分析研究十分有限且缺少针对性。为了研究本草香颗粒态PAHs的排放因子和排放特征,在自制的试验舱内采集5种本草香燃烧后的颗粒物,通过优化萃取溶剂、超声时间和仪器分析条件,建立了超声提取-气相色谱-质谱(GC-MS)测定本草香燃烧后颗粒物上所吸附的16种PAHs的方法。通过采集0.8 g样品,切取整片滤膜样品,使用正己烷-二氯甲烷(1∶1, v/v)进行超声萃取,经浓缩定容过滤后使用气相色谱-质谱分析,内标法定量。结果表明,16种PAHs在0.1~5.0 μg/mL范围内线性良好(相关系数*r*^2^>0.998),方法检出限(MDL)为0.4~3.8 ng/g;低、高2个水平的加标回收率分别为77.4%~99.5%和82.0%~101.3%;相对标准偏差(RSD)为0.7%~7.2%。5种本草香颗粒态PAHs的排放因子为4.60~11.89 μg/g。本草香的16种颗粒态PAHs中菲(Phe)的含量均为最高,所占比例为24.85%~42.59%,其次为荧蒽(Flu)、芘(Pyr)、 $\overset{艹}{屈}$ (Chr)、蒽(Ant)。本草香颗粒态PAHs中Phe的含量稳定且占比明显高于其他室内燃烧源,可将Phe作为本草香的颗粒态特征PAHs。颗粒态PAHs主要分布在3环和4环上,3环和4环PAHs占比之和为83.84%~96.31%。颗粒态的Phe/Flu比值可作为辨别不同室内燃烧源中燃香释放源的特征比值。该方法所需样品量少,灵敏度高,前处理操作简便,适用于燃香类产品中PAHs的快速检测,同时为了解本草香颗粒态PAHs分布规律和健康危害提供科学数据。

## Abstract (en)

Polycyclic aromatic hydrocarbons (PAHs) have attracted global attention because they are carcinogens and mutagenic to humans. To date, more than 200 PAHs have been found. The United States Environmental Protection Agency (USEPA) has designated 16 PAH species as priority control pollutants due to their highly toxic substances. Herbal incense is frequently used in daily life. As a result, it is critical to investigate its impact on human health and environmental safety. However, research on particle-phase PAHs is very limited and inapplicable. The current research focuses mainly on bamboo incense, which has a simpler formula than herbal incense.

In this study, the emission factor and emission characteristics of particle-phase PAHs from herbal incense were described. A method combining ultrasonic and gas chromatography-mass spectrometry (GC-MS) was developed for the simultaneous determination of 16 particle-phase PAHs of herbal incense. The settings for extraction solvent, ultrasonic time, and instrument analysis conditions were optimized. In the test chamber, samples were collected by burning 0.8 g of herbal incense. After combustion, PAHs adsorbing on the particles of herbal incense were collected on a quartz filter. The whole filter sample was sliced and extracted with *n*-hexane-dichloromethane (1∶1, v/v). A rotary evaporator was used to concentrate the extract. GC-MS was used to analyze the prepared sample. The internal standard method was used to perform quantitative analysis on the target compounds. The linearities of the 16 target PAHs were good between mass concentrations of 0.1-5.0 μg/mL, with correlation coefficients greater than 0.998. The method detection limits (MDLs) of the 16 PAHs ranged from 0.4 to 3.8 ng/g. The 0.625 μg/g and 1.25 μg/g spiked recoveries ranged from 77.4% to 99.5% and 82.0% to 101.3%, respectively. The relative standard deviations (RSDs) of the 16 PAHs were ranged from 0.7% to 7.2% (*n*=6). The emission factors of particle-phase PAHs from five different kinds of herbal incense ranged from 4.60 to 11.89 μg/g. The highest concentration of phenanthrene (Phe) was found in 16 particle-phase individual PAHs of herbal incense. Fluoranthene (Flu), pyrene (Pyr), chrysene (Chr) and anthracene (Ant) concentration were ranked after Phe. The sum of these five proportions was 73.00%-89.97%. The proportion of Phe in herbal incense particle-phase PAHs was significantly higher than that of other indoor combustion sources. As a result, Phe could be used to identify individual PAHs in the particle-phase of herbal incense. The particle-phase PAHs were mainly distributed on the 3-ring and 4-ring, with a sum of 83.84% to 96.31% on the 3-ring and 4-ring. The proportion of high-molecular weight PAHs in the samples ranged from 44.25% to 63.31%. The proportion of low-molecular weight PAHs in the samples ranged from 36.69% to 55.75%. The incense source could be distinguished from other indoor combustion sources by its distinctive Phe/Flu ratio. The established method has high sensitivity, simple operation, and requires fewer samples. This method is suitable for rapidly detecting PAHs in burning incense. At the same time, it provides scientific data for further studies on the distribution and health effects of particle-phase PAHs of herbal incense.

本草香是以中草药粉末为原料,遵循中医方剂学“君、臣、佐、使”的原则进行配伍、炮制,同时加以植物粘合剂制成的香品^[1,2]^。本草香点燃后释放的烟气通过口、鼻、皮肤接触等途径对人起到调节功效,但同时也会产生颗粒物、一氧化碳等环境污染物^[3]^。多环芳烃(PAHs)是一类由2个或2个以上芳香环组成的有机物,易在颗粒物上富集,通过呼吸系统进入人体。其中,被美国环境保护署(USEPA)列为优先控制的16种PAHs因其具有致癌性、生物难降解性和累积性而备受关注^[4⇓-6]^。目前,PAHs的检测对象主要集中在土壤、水、食品和空气等^[7]^,针对香品燃烧产生PAHs的含量测定的研究报道十分有限。已有的研究主要对象为竹签香,竹签香多用于寺庙、佛堂等场所,而本草香主要用于居室,与人们日常生活更为相关。因此,研究本草香颗粒态PAHs对人体健康和环境安全的影响十分必要。

本文采用超声提取-气相色谱-质谱法,建立了测定本草香中16种颗粒态多环芳烃的高灵敏分析方法。该方法所需样品量少,样品提取和制备简单,具有较高的准确度和灵敏性。

## 1 实验部分

### 1.1 仪器、试剂与材料

7890B-5977B气相色谱-质谱联用仪(EI源,美国Agilent公司); KQ-500DE超声波清洗器(昆山市超声仪器有限公司); RE-52AA旋转蒸发仪(上海亚荣生化仪器厂); 81.2 L自制试验舱(直径30 cm,高度115 cm),示意图见图1。

**图1:**
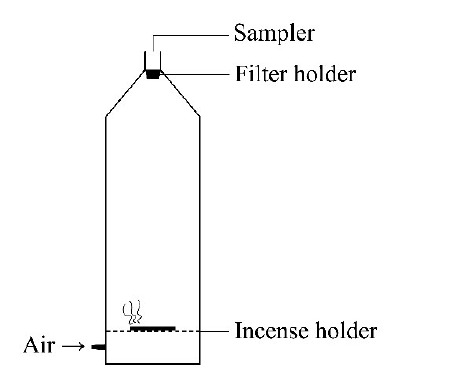
试验舱示意图

16种多环芳烃混合标准溶液、5种多环芳烃同位素内标混合标准溶液质量浓度均为2000 mg/L,购自美国O2si公司;石英滤膜(直径37 mm)、聚四氟乙烯过滤器(孔径0.45 μm)均购自英国Whatman公司;二氯甲烷、正己烷均为色谱纯,购自德国CNW公司。

选取市售的5种本草香作为研究对象,分别编号为S1~S5,具体样品信息见表1。

**表1:** 样品信息

No.	Type	Shape	Specification	Single weight/g	Moisture rate/%^[8]^	Mass loss during combustion/%
S1	herbal smell incense	incense coil	48 discs	2.35	7.70	86.5
S2	herbal smell incense	incense stick	21 cm	0.45	7.61	78.0
S3	herbal smell incense	incense stick	21 cm	0.42	8.76	88.4
S4	sandalwood incense	incense coil	40 discs	16.65	8.28	89.6
S5	eaglewood incense	incense stick	21 cm	0.85	8.96	93.1

### 1.2 实验方法

#### 1.2.1 标准工作溶液的配制

以正己烷为溶剂,将16种PAHs混合标准溶液稀释成50 μg/mL的标准储备液;5种PAHs同位素内标混合标准溶液稀释成50 μg/mL的内标使用液。采用正己烷稀释标准储备液,制备质量浓度为0.1~5.0 μg/mL的PAHs系列标准工作溶液,待用。

#### 1.2.2 样品采集

采样前,擦拭舱内表面并通风10 min以上,以降低舱内本底干扰。取0.8 g(精确至0.001 g)样品点燃后开启采样仪,采集流量为10 L/min,燃烧结束后继续采集20 min。石英滤膜使用前需置于450 ℃马弗炉中焙烧4 h,降低有机杂质的干扰;采样结束后,经24 h恒温干燥后称重,记录采样前后滤膜重量。

#### 1.2.3 样品前处理

将采样后的石英滤膜剪成小块,装入样品瓶中,加入10 mL正己烷-二氯甲烷(1∶1, v/v)超声萃取30 min,将提取液转移到50 mL圆底烧瓶中,重复上述步骤一次并合并提取液。于33 ℃旋转蒸发浓缩至1 mL左右,加入5 mL正己烷,继续浓缩至1 mL以下,加入8 μL 50 μg/mL的内标使用液,用正己烷定容至1 mL,过滤器将滤液过滤至样品瓶。

#### 1.2.4 色谱-质谱条件及分析

色谱柱:HP-5MS毛细管柱(30 m×0.25 mm×0.25 μm);进样口温度:290 ℃;流速:1.2 mL/min;进样量:1 μL;进样方式:不分流进样;柱温升温程序:50 ℃保持5 min,以10 ℃/min速率升到260 ℃,再以6 ℃/min速率升到300 ℃,保持6 min。

离子源温度:230 ℃;传输线温度:280 ℃;四极杆温度:150 ℃;测定模式:选择离子监测模式;溶剂延迟时间:11 min;根据PAHs的相对保留时间和特征离子定性,根据定量离子峰面积采用内标法定量,见表2。

**表2:** 16种多环芳烃和5种内标的GC-MS参数

PAH	Retention time/min	Ion pairs (m/z)	IS
Naphthalene-D_8_(Nap-D_8_)	12.841	136^*^, 137, 68	
Naphthalene (Nap)	12.885	128^*^, 129, 127	Nap-D_8_
Acenaphthylene (AcPy)	16.723	152^*^, 153, 151	AcP-D_10_
Acenaphthene-D_10_(AcP-D_10_)	17.098	164^*^, 162	
Acenaphthene (AcP)	17.171	154^*^, 153, 152	AcP-D_10_
Fluorene (Fluo)	18.377	166^*^, 167, 165	Phe-D_10_
Phenanthrene-D_10_(Phe-D_10_)	20.570	188^*^, 94	
Phenanthrene (Phe)	20.625	178^*^, 179, 176	Phe-D_10_
Anthracene (Ant)	20.731	178^*^, 179, 176	Phe-D_10_
Fluoranthene (Flu)	23.458	202^*^, 203, 101	Phe-D_10_
Pyrene (Pyr)	23.963	202^*^, 203, 101	Phe-D_10_
Benzo[a]anthracene (BaA)	26.871	228^*^, 229, 226, 114	Chr-D_12_
Chrysene-D_12_(Chr-D_12_)	26.907	240^*^, 241, 120	
Chrysene (Chr)	26.970	228^*^, 229, 226, 114	Chr-D_12_
Benzo[b]fluoranthene (BbF)	29.677	252^*^, 253, 126	Per-D_12_
Benzo[k]fluoranthene (BkF)	29.742	252^*^, 253, 126	Per-D_12_
Benzo[a]pyrene (BaP)	30.496	252^*^, 253. 126	Per-D_12_
Perylene-D_12_(Per-D_12_)	30.501	264^*^, 265, 260	
Indeno[1,2,3-cd]pyrene (InP)	33.436	276^*^, 227, 138	Per-D_12_
Dibenz[a,h]anthracene (DBA)	33.539	278^*^, 279. 139	Per-D_12_
Benzo[ghi]perylene (BghiP)	34.135	276^*^, 277. 138	Per-D_12_

* Quantitative ion.

通过GC-MS分析得到的PAHs质量和燃烧质量计算本草香颗粒态PAHs的排放因子(*F*,μg/g),计算公式如下:


(1) *F*=*ρV/m*

式中,*ρ*为目标物的质量浓度(μg/mL); *V*为样品的浓缩体积(mL); *m*为样品的燃烧质量(g)。

## 2 结果与讨论

### 2.1 提取溶剂的选择

PAHs是非极性物质,通常采用正己烷、二氯甲烷、丙酮或混合溶剂等进行提取^[7,9]^。本文采用正己烷、二氯甲烷、正己烷-二氯甲烷(1∶1, v/v)考察其对PAHs提取效率的影响。结果如图2所示,正己烷-二氯甲烷(1∶1, v/v)、正己烷和二氯甲烷提取目标化合物的回收率分别为87.4%~102.2%、73.4%~115.5%和71.1%~103.3%。与正己烷、二氯甲烷提取体系的回收率相比,正己烷-二氯甲烷(1∶1, v/v)的回收率更高。因此,本文选择正己烷-二氯甲烷(1∶1, v/v)作为提取溶剂。

**图2:**
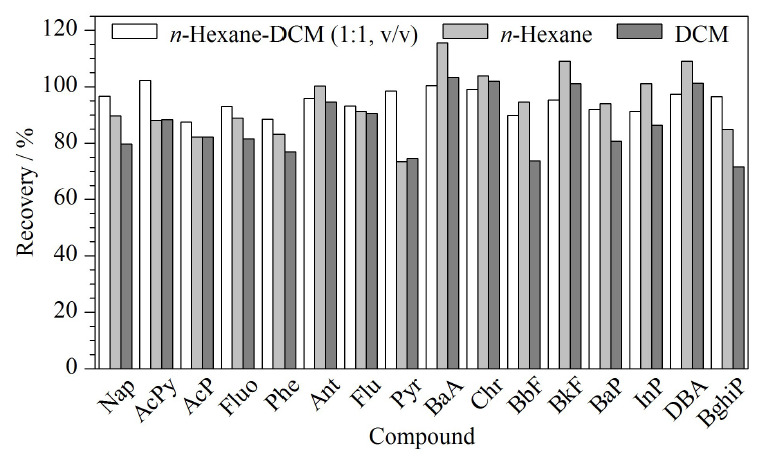
不同提取溶剂对本草香中16种颗粒态PAHs 回收率的影响

### 2.2 提取时间的选择

以正己烷-二氯甲烷(1∶1, v/v)为提取溶剂,考察不同超声时间(15、30、45 min)对PAHs提取效率的影响。结果如图3所示,超声时间15 min的目标化合物回收率为45.3%~109.8%, 8个化合物回收率小于80%;超声时间30 min的目标化合物回收率为73.8%~104.1%,只有Nap和AcP的回收率低于80%;超声时间45 min的目标化合物回收率为70.4%~114.9%,除了Nap、AcP之外,还有BkF和BghiP共4个化合物回收率低于80%。除了AcP、Ant、BaA、DBA 4个化合物的回收率在45 min时略高于30 min,其余目标化合物的提取效率在30 min达到最佳。因此综合考虑16种PAHs的提取效率,本文选择超声时间为30 min。

**图3:**
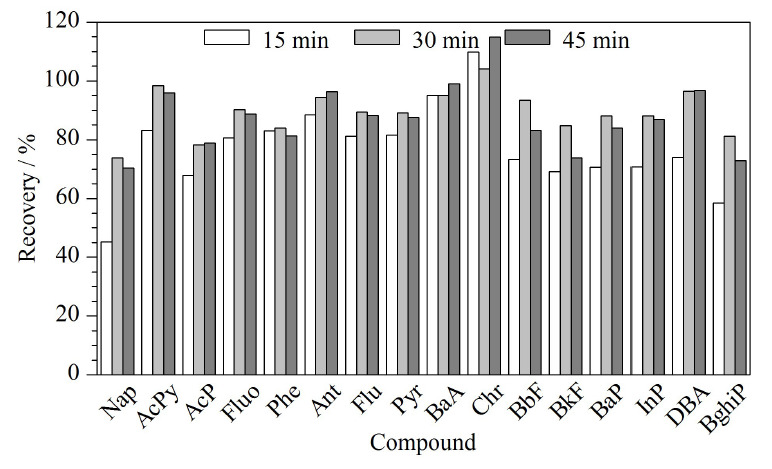
超声时间对本草香16种颗粒态PAHs回收率的影响

### 2.3 方法学评价

在优化条件下,配制0.1、0.2、0.5、1.0、2.0、5.0 μg/mL的系列混合标准工作液,以峰面积(*y*)为纵坐标,质量浓度(*x*, μg/mL)为横坐标,绘制标准曲线。16种PAHs在0.1~5.0 μg/mL范围内线性良好,相关系数(*r*^2^)均≥0.998。平行测定11份空白加标(100 ng/mL)样品,计算11次结果的标准偏差(*S*)。方法检出限(MDL)=*t*_(_*_n_*_-1, 0.99)_×*S*,式中*n*为平行测定的次数;*t*_(_*_n_*_-1, 0.99)_为自由度为*n*-1、置信度为0.99时的*t*分布值(单侧),参考HJ 168-2010^[10]^,当*n*=11时,*t*_(_*_n_*_-1, 0.99)_为2.764,通过计算,16种PAHs的MDL为0.4~3.8 ng/g。取一个样品分别进行2个水平的加标回收试验,重复3次,并分别测定6份低水平的加标样品,计算相对标准偏差(RSD)。除了Nap和AcP的低水平回收率低于80%外,其余14种PAHs的回收率为82.0%~101.3%, RSD为0.7%~7.2%,结果见表3。

**表3:** 16种PAHs的回归方程、相关系数、方法检出限、回收率和相对标准偏差

PAH	Regression equation	r^2^	MDL/(ng/g)	Recoveries (n=3)/%		RSD (n=6)/%
				0.625 μg/g	1.25 μg/g	
Nap	Y=0.913X+0.196	0.999	2.7	78.6	94.5	1.5
AcPy	Y=1.613X+0.269	0.999	2.4	97.8	98.4	3.7
AcP	Y=1.033X+0.207	0.998	3.7	77.4	92.4	1.4
Fluo	Y=1.274X+0.294	0.998	3.4	93.6	99.0	1.6
Phe	Y=0.930X+0.258	0.999	3.3	85.7	94.9	1.0
Ant	Y=0.868X+0.138	0.998	2.9	98.0	95.4	4.8
Flu	Y=1.046X+0.260	0.998	3.1	95.2	96.8	1.7
Pyr	Y=1.055X+0.258	0.998	3.8	94.9	96.4	1.2
BaA	Y=1.040X+0.070	0.999	0.4	99.5	101.3	6.4
Chr	Y=0.686X+0.109	0.998	0.4	91.6	99.0	0.7
BbF	Y=1.260X+0.176	0.998	0.9	97.7	82.8	1.6
BkF	Y=1.253X+0.108	0.999	0.9	90.6	82.0	2.8
BaP	Y=1.070X-0.049	0.999	0.7	89.5	85.7	6.8
InP	Y=0.864X-0.029	0.999	0.7	88.1	85.6	7.2
DBA	Y=0.745X+0.046	0.999	2.3	91.8	84.1	5.8
BghiP	Y=0.965X+0.124	0.999	3.1	86.2	84.7	3.1

*Y*: peak area; *X*: mass concentration, μg/mL.

### 2.4 本草香颗粒态PAHs的排放因子

应用本方法对5种市售本草香燃烧后颗粒物中16种PAHs进行测定,5个样品的颗粒态PAHs排放因子为4.60~11.89 μg/g,其中S2的PAHs排放因子最大,为11.89 μg/g, S3的PAHs排放因子最小,为4.60 μg/g, S2的PAHs排放因子是S3的2.6倍(见表4)。张剑辉等^[11]^测得两种竹签香的颗粒态PAHs排放因子分别为10.52 μg/g和13.79 μg/g; Yang等^[12]^测得9种竹签香的颗粒态PAHs排放因子为4.5~6.9 μg/g;张金萍等^[13]^对线香和竹签香燃烧释放的PM_10_中PAHs进行检测,得到排放因子为0.79~3.44 μg/g,线香的排放因子大于竹签香;Yang等^[14]^对有烟、微烟和无烟竹签香测得PM_2.5_中PAHs的排放因子分别为8.78、3.54和1.85 μg/g。虽然这些报道研究了燃香不同颗粒物粒径上PAHs的排放因子,但燃香燃烧释放的颗粒物中,95%以上的颗粒物粒径小于1.0 μm^[12]^。因此,PM_2.5_和PM_10_中PAHs的排放因子与总悬浮颗粒物(TSP)中PAHs的含量相差较小。综上,本文5种本草香中颗粒态PAHs的排放因子虽然相差略大,但总体与已报道的数值相符。而有些竹签香颗粒态PAHs排放因子较低,可能是因为制香过程中添加香精、碳粉和滑石粉等,减少了天然香料含量的使用,从而降低了TSP及其吸附的PAHs的释放量。值得注意的是,S2燃烧前后质量损失率最低(78.0%),但PAHs排放因子最大,这可能是不同香品中原材料和配比的差异性造成的,因为香品中低的碳含量会减少颗粒态PAHs的排放因子^[12,15]^。

**表4:** 本草香颗粒态PAHs的排放因子

PAH	Emission factors/(μg/g)				
	S1	S2	S3	S4	S5
Nap	ND	ND	ND	ND	0.05
AcPy	0.34	0.21	0.02	0.15	0.26
AcP	ND	ND	ND	ND	ND
Fluo	0.11	0.21	0.15	0.02	0.15
Phe	3.50	4.83	1.75	2.50	2.21
Ant	0.92	1.38	0.24	0.61	0.60
Flu	1.67	2.00	0.79	0.96	1.33
Pyr	1.20	1.42	0.58	0.74	0.99
BaA	0.68	0.32	0.24	0.21	0.56
Chr	1.63	1.01	0.51	0.48	1.37
BbF	0.31	0.13	0.07	0.06	0.32
BkF	0.17	0.07	0.03	ND	0.21
BaP	0.39	0.19	0.13	0.13	0.34
InP	0.25	0.10	0.08	0.03	0.24
DBA	0.14	0.02	0.01	ND	0.13
BghiP	0.08	ND	ND	ND	0.15
∑PAHs	11.39	11.89	4.60	5.88	8.90

ND: below the MDL.

### 2.5 本草香颗粒态PAHs的分布特征

16种PAHs化合物致癌性存在差异,明确本草香颗粒态PAHs的分布特征,可以在燃香对人体的健康危害和环境的减排控制方面起到重要参考作用^[15,16]^。从表5可以看出,在16种PAHs中,Phe的含量在5种本草香中均为最高,所占比例为24.85%~42.59%,其中S4中比例最高,为42.59%。其次为Flu、Pyr、Chr和Ant,这5种个体PAHs在16种PAHs中占比之和为73.00%~89.97%,本结果与张金萍等^[13]^的结论接近,也符合Phe、Flu、Pyr作为木材燃烧标识物的报道^[19]^。除了AcP在5个样品中均未检出之外,其余15种PAHs在5个样品均有检出或部分检出。强致癌的BaP在样品中均有检出,占比为1.60%~3.85%,不同室内燃烧源的BaP含量在16种PAHs占比均靠中。在室内不同燃烧源中薪柴和燃香的Phe含量占比都是最高的,但与薪柴相比,燃香中Phe含量具有明显优势,且占比均大于25.0%,高于其他室内燃烧源,说明Phe在燃香释放的颗粒态PAHs中含量高且相对稳定,可作为燃香的颗粒态特征PAH。

**表5:** 不同室内燃烧源中16种PAHs的含量百分比

PAH	Content percentages/%														
	S1	S2	S3	S4	S5	Incense stick^[13]^	Bamboo incense^[13]^	Mosquito coil incense^[11]^	Cigarette^[11]^	Firewood^[17]^	Straw^[17]^		Coal^[18]^
Nap	0	0	0	0	0.52	2.15	2.13	0.10	0.46	0.51	0.52		3.34
AcPy	3.00	1.76	0.47	2.49	2.93	1.68	1.36	0.01	0.17	0.31	0.31		1.14
AcP	0	0	0	0	0	1.48	1.96	0.08	0.77	0.15	0.01		1.41
Fluo	0.97	1.79	3.31	0.30	1.67	4.07	2.81	0.05	0.57	2.02	2.28		6.11
Phe	30.74	40.62	38.12	42.59	24.85	30.18	27.11	1.88	7.11	10.64	19.41		12.20
Ant	8.06	11.58	5.26	10.35	6.72	8.05	10.66	1.14	4.19	1.88	2.79		4.05
Flu	14.64	16.79	17.14	16.29	14.93	13.80	14.92	3.97	12.30	20.28	13.90		12.73
Pyr	10.52	11.96	12.65	12.66	11.14	10.02	14.49	6.24	15.40	18.36	13.99		10.36
BaA	6.01	2.70	5.27	3.54	6.25	5.52	4.09	16.39	17.55	7.35	6.89		10.73
Chr	14.29	8.50	11.05	8.09	15.36	6.97	5.54	8.07	14.32	8.16	9.27		7.44
BbF	2.72	1.09	1.40	0.96	3.61	2.00	1.36	8.74	3.78	8.14	9.63		8.10
BkF	1.52	0.62	0.56	0	2.34	2.50	0.94	14.11	4.98	3.58	3.69		2.19
BaP	3.44	1.60	2.88	2.18	3.85	3.19	2.90	10.28	8.79	6.05	6.65		4.30
InP	2.17	0.81	1.74	0.48	2.68	3.05	3.41	11.64	5.03	6.86	4.42		6.52
DBA	1.25	0.19	0.17	0.07	1.49	2.41	3.24	15.15	3.39	1.12	1.79		4.33
BghiP	0.66	0	0	0	1.67	2.93	3.07	2.15	1.19	4.60	4.45		5.05

随着芳香环数和相对分子质量的增加,PAHs的性质和结构也会变化^[20]^。将16种PAHs按所含芳香环数进行分类,分为2环(Nap)、3环(AcPy, AcP, Fluo, Phe, Ant)、4环(Flu, Pyr, BaA, Chr)、5环(BbF, BkF, BaP, DBA)和6环(InP, BghiP)。5种本草香颗粒态PAHs环数中,3环和4环占主导,3环和4环占比之和为83.84%~96.31%。其中,S2的3环占比最高为55.75%, 4环占比最低为39.95%。S5的4环占比最高为47.67%, 3环占比最低为36.17%。其中,2~3环的PAHs被认为相对分子质量较低(LMW), 4~6环被认为相对分子质量较高(HMW),一般HMW PAHs的致癌性会高于LMW,但2环的Nap对人的致癌性比一些HMW PAHs还高^[16,21]^。本文中,HMW PAHs占比为44.25%~63.31%, LMW PAHs占比为36.69%~55.75%, S5的颗粒态HMW PAHs释放量最高。2环的Nap只在S5中有检出,且占比仅为0.52%,这是因为2环主要存在于气相中,结果见图4。

**图4:**
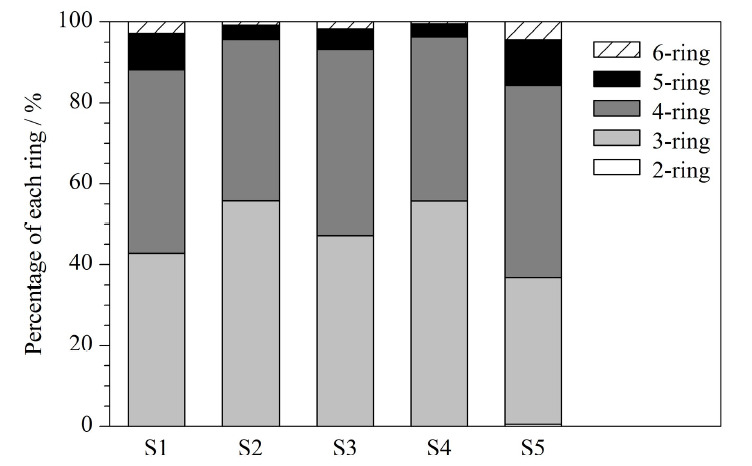
本草香颗粒态PAHs的环数分布

由上可知,本草香颗粒态PAHs中Phe、Flu、Pyr、Chr、Ant是主要个体,其中Phe是本草香的颗粒态特征PAH,分布在3环和4环上。

### 2.6 本草香颗粒态PAHs的特征比值

PAHs异构体的浓度比值相对比较稳定,常被作为特征比值用于判断PAHs的释放源^[22]^。本文将不同燃香种类和其他室内燃烧源的排放比值进行对比,判断属于燃香释放源的特征比值,结果见表6。Flu/(Flu+Pyr)、InP/(InP+BghiP)、BaA/(BaA+Chr)和Ant/(Ant+Phe)具有较好的稳定性被广泛用于释放源的定性辨别^[23,24]^,本草香的4个特征比值分别是0.58、0.88、0.29、0.19, Flu/(Flu+Pyr)的比值在不同室内燃烧源中相似;InP/(InP+BghiP)均大于0.5,本草香与张剑锋等^[11]^报道的蚊香和香烟数值更接近;BaA/(BaA+Chr)的比值在不同室内燃烧源差别较大;本草香Ant/(Ant+Phe)比值与张金萍等^[13]^报道的香品、段升飞等^[17]^的木材和张宜升等^[18]^的煤炭数值接近。因此,当室内存在多个燃烧源时,无法从这4个特征比值区分出燃香源。张金萍等^[13]^通过Phe/Ant和Pyr/BaP比值来区分木材和燃香源,本草香中这两个比值分别为4.48、4.72,木材分别为5.67、3.04, Phe/Ant比值虽然可以区分燃香和木材、蚊香、香烟、秸秆,但比值在不同燃香种类中相差较大,且张金萍等^[13]^报道的线香比值与煤炭接近;本草香和线香的Pyr/BaP比值接近且高于其他室内燃烧源,但张金萍等^[13]^报道的竹签香比值明显小于本草香和线香,且与木材比值相似。通过Phe/Ant和Pyr/BaP比值也无法有效判断燃香源。本文引入Phe/Flu比值,发现本草香、线香和竹签香的比值均接近2.00,且明显区别于其他室内燃烧源,因此可以通过Phe/Flu比值区分燃香源与其他室内燃烧源。但本草香和其他燃香种类主要成分的来源差异小,因此难以用特征比值来区分。同时,燃烧条件以及PAHs排放到环境后存在转化行为等均会影响特征比值,而且本实验燃香样本数有限,因此以上结论还需进一步研究。

**表6:** 本草香颗粒态PAHs的特征比值

Item	Herbal incense (this study)	Incense stick^[13]^	Bamboo incense^[13]^	Mosquito coil incense^[11]^	Cigarette^[11]^	Firewood^[17]^	Straw^[17]^	Coal^[18]^
Flu/(Flu+Pyr)	0.58	0.51	0.58	0.39	0.44	0.52	0.50	0.55
InP/(InP+BghiP)	0.88	0.53	0.51	0.84	0.81	0.60	0.50	0.56
BaA/(BaA+Chr)	0.29	0.42	0.44	0.67	0.55	0.47	0.43	0.59
Ant/(Ant+Phe)	0.19	0.28	0.21	0.38	0.37	0.15	0.13	0.25
Phe/Ant	4.48	2.54	3.75	1.65	1.70	5.67	6.96	3.01
Pry/BaP	4.72	5.00	3.14	0.61	1.75	3.04	2.10	2.41
Phe/Flu	2.21	1.82	2.19	0.47	0.58	0.52	1.40	0.96

## 3 结论

本研究通过对本草香燃烧后采集的颗粒物进行超声萃取,对萃取过程中的相关参数进行优化,萃取液经浓缩定容过滤后采用GC-MS对16种PAHs进行分析,建立了一种快速检测燃香类产品16种颗粒态PAHs的分析方法。该方法前处理简单,只需要有机溶剂萃取和滤膜过滤,具有快速简便、准确度高和精密度好的优点。同时,对本草香16种颗粒态PAHs的分布特征进行分析,了解了本草香颗粒态的PAHs分布规律和对人体潜在的健康危害。对比不同燃香种类和其他室内燃烧源的排放比值,确定了燃香释放源的特征比值。将该分析方法进一步扩展,可以为其他燃香种类的PAHs研究提供参考,为燃香的质量控制提供科学数据。
